# Supplementary material for: Modeling glioblastoma heterogeneity as a dynamic network of cell states
Source: Mol Syst Biol. 2021 Sep 16;17(9):e10105. doi: 10.15252/msb.202010105 (PMC8444284; doi:10.15252/msb.202010105)
Supplement: Supplementary file 5 — Source Data for Figure 3 [file MSB-17-e10105-s001.zip › Figure3A_sourcedata/GSEA_3065/hallmarks_state5.GseaPreranked.1623416302988/HALLMARK_CHOLESTEROL_HOMEOSTASIS.html]

Details for gene set HALLMARK\_CHOLESTEROL\_HOMEOSTASIS[GSEA]

|  || Dataset | state5 |
| Phenotype | NoPhenotypeAvailable |
| Upregulated in class | na\_pos |
| GeneSet | HALLMARK\_CHOLESTEROL\_HOMEOSTASIS |
| Enrichment Score (ES) | 0.4038337 |
| Normalized Enrichment Score (NES) | 1.4915272 |
| Nominal p-value | 0.017985612 |
| FDR q-value | 0.07203621 |
| FWER p-Value | 0.161 |
Table: GSEA Results Summary

  

Fig 1: Enrichment plot: HALLMARK\_CHOLESTEROL\_HOMEOSTASIS      
 Profile of the Running ES Score & Positions of GeneSet Members on the Rank Ordered List

  

| PROBE | GENE SYMBOL | GENE\_TITLE | RANK IN GENE LIST | RANK METRIC SCORE | RUNNING ES | CORE ENRICHMENT || 1 | CD9 |  |  | 14 | 0.857 | 0.0599 | Yes |
| 2 | SQLE |  |  | 39 | 0.705 | 0.1078 | Yes |
| 3 | HMGCR |  |  | 79 | 0.562 | 0.1438 | Yes |
| 4 | GUSB |  |  | 143 | 0.489 | 0.1718 | Yes |
| 5 | CLU |  |  | 162 | 0.474 | 0.2038 | Yes |
| 6 | ALCAM |  |  | 225 | 0.422 | 0.2271 | Yes |
| 7 | SCD |  |  | 284 | 0.391 | 0.2486 | Yes |
| 8 | TM7SF2 |  |  | 352 | 0.360 | 0.2670 | Yes |
| 9 | DHCR7 |  |  | 387 | 0.347 | 0.2881 | Yes |
| 10 | LDLR |  |  | 404 | 0.339 | 0.3106 | Yes |
| 11 | TNFRSF12A |  |  | 615 | 0.282 | 0.3073 | Yes |
| 12 | LPL |  |  | 646 | 0.276 | 0.3237 | Yes |
| 13 | FADS2 |  |  | 780 | 0.245 | 0.3264 | Yes |
| 14 | ATXN2 |  |  | 808 | 0.239 | 0.3405 | Yes |
| 15 | GPX8 |  |  | 874 | 0.229 | 0.3497 | Yes |
| 16 | SREBF2 |  |  | 882 | 0.228 | 0.3652 | Yes |
| 17 | TMEM97 |  |  | 995 | 0.211 | 0.3678 | Yes |
| 18 | EBP |  |  | 1041 | 0.206 | 0.3775 | Yes |
| 19 | CYP51A1 |  |  | 1061 | 0.203 | 0.3900 | Yes |
| 20 | SC5D |  |  | 1094 | 0.199 | 0.4007 | Yes |
| 21 | JAG1 |  |  | 1304 | 0.170 | 0.3895 | Yes |
| 22 | GLDC |  |  | 1341 | 0.167 | 0.3974 | Yes |
| 23 | ANTXR2 |  |  | 1389 | 0.163 | 0.4038 | Yes |
| 24 | SEMA3B |  |  | 1604 | 0.141 | 0.3900 | No |
| 25 | HMGCS1 |  |  | 1899 | 0.118 | 0.3655 | No |
| 26 | ABCA2 |  |  | 1975 | 0.113 | 0.3652 | No |
| 27 | LGMN |  |  | 2091 | 0.106 | 0.3600 | No |
| 28 | PLAUR |  |  | 2826 | 0.061 | 0.2821 | No |
| 29 | FDPS |  |  | 2849 | 0.060 | 0.2840 | No |
| 30 | HSD17B7 |  |  | 3006 | 0.051 | 0.2702 | No |
| 31 | LSS |  |  | 3145 | 0.044 | 0.2579 | No |
| 32 | FASN |  |  | 3225 | 0.040 | 0.2519 | No |
| 33 | CHKA |  |  | 3386 | 0.032 | 0.2363 | No |
| 34 | CTNNB1 |  |  | 3417 | 0.031 | 0.2351 | No |
| 35 | NSDHL |  |  | 3526 | 0.026 | 0.2249 | No |
| 36 | CXCL16 |  |  | 3689 | 0.020 | 0.2082 | No |
| 37 | MVD |  |  | 4194 | -0.001 | 0.1518 | No |
| 38 | ATF3 |  |  | 4225 | -0.002 | 0.1486 | No |
| 39 | MVK |  |  | 4385 | -0.010 | 0.1315 | No |
| 40 | GNAI1 |  |  | 4664 | -0.021 | 0.1018 | No |
| 41 | CPEB2 |  |  | 4705 | -0.022 | 0.0989 | No |
| 42 | ACSS2 |  |  | 4719 | -0.023 | 0.0991 | No |
| 43 | PLSCR1 |  |  | 5043 | -0.034 | 0.0653 | No |
| 44 | ERRFI1 |  |  | 5451 | -0.050 | 0.0233 | No |
| 45 | TP53INP1 |  |  | 5454 | -0.050 | 0.0267 | No |
| 46 | STX5 |  |  | 6536 | -0.104 | -0.0869 | No |
| 47 | ATF5 |  |  | 6540 | -0.104 | -0.0798 | No |
| 48 | PCYT2 |  |  | 7182 | -0.154 | -0.1406 | No |
| 49 | PMVK |  |  | 7311 | -0.168 | -0.1429 | No |
| 50 | IDI1 |  |  | 7337 | -0.172 | -0.1333 | No |
| 51 | ETHE1 |  |  | 7475 | -0.189 | -0.1351 | No |
| 52 | FDFT1 |  |  | 7852 | -0.244 | -0.1598 | No |
| 53 | PNRC1 |  |  | 7867 | -0.247 | -0.1436 | No |
| 54 | ECH1 |  |  | 8238 | -0.335 | -0.1610 | No |
| 55 | ACAT2 |  |  | 8440 | -0.404 | -0.1546 | No |
| 56 | LGALS3 |  |  | 8550 | -0.460 | -0.1338 | No |
| 57 | ANXA5 |  |  | 8626 | -0.510 | -0.1057 | No |
| 58 | S100A11 |  |  | 8767 | -0.626 | -0.0765 | No |
| 59 | FABP5 |  |  | 8829 | -0.684 | -0.0343 | No |
| 60 | ACTG1 |  |  | 8859 | -0.718 | 0.0140 | No |
Table: GSEA details [plain text format]

  

Fig 2: HALLMARK\_CHOLESTEROL\_HOMEOSTASIS: Random ES distribution      
 Gene set null distribution of ES for **HALLMARK\_CHOLESTEROL\_HOMEOSTASIS**
